# Structured Functional Assessment Pathway and Pharmacological Optimization During Cardiovascular Rehabilitation in Chronic Heart Failure: A Retrospective Tertiary Center Study

**DOI:** 10.3390/life16040603

**Published:** 2026-04-04

**Authors:** Miruna Popovici, Abhinav Sharma, Gabriel Florin Razvan Mogos, Nilima Rajpal Kundnani, Daniel Duda Marius Seiman, Victor Buciu, Simona Ruxanda Dragan

**Affiliations:** 1 Doctoral School, “Victor Babes” University of Medicine and Pharmacy, E. Murgu Square, No. 2, 300041 Timisoara, Romania; 2 Department VI-Cardiology, “Victor Babes” University of Medicine and Pharmacy, 300041 Timisoara, Romania; 3 Research Centre of Timisoara Institute of Cardiovascular Diseases, “Victor Babes” University of Medicine and Pharmacy, 300041 Timisoara, Romania; 4 Department of Surgery, University of Medicine and Pharmacy of Craiova, 200349 Craiova, Romania

**Keywords:** heart failure, cardiovascular rehabilitation, 6MWT, GDMT, cardiopulmonary exercise testing

## Abstract

Introduction: Optimization of guideline-directed medical therapy in chronic heart failure remains challenging in real-world practice, particularly outside settings with routine cardiopulmonary exercise testing. In this context, cardiovascular rehabilitation can improve functional capacity, symptoms, and quality of life, while structured follow-up may also facilitate treatment adjustment. We therefore evaluated whether exposure to a structured multimodal functional assessment pathway, embedded within a more intensive follow-up model, was associated with pharmacological optimization and functional change in chronic heart failure. Methods: We conducted a retrospective, single-center cohort study including adults with chronic heart failure with reduced or mildly reduced ejection fraction managed in a tertiary university clinic. Patients were classified according to documented exposure to an integrated pathway that combined standardized 6 min walk testing, heart rate dynamics, oxygen saturation response, perceived exertion, validated quality-of-life assessment, and prespecified interim reassessment, versus usual care. The integrated pathway involved more frequent clinical contact than usual care. The primary outcome was change in 6 min walk distance over 6 months. Secondary outcomes included changes in heart rate recovery, oxygen saturation nadir, Borg perceived exertion score, quality-of-life score, intensity of guideline-directed medical therapy, treatment intensification rates, and heart failure hospitalization. Results: The study included 250 patients with comparable baseline demographic and clinical characteristics. Patients managed within the structured pathway showed greater improvement in 6 min walk distance at 6 months than those receiving usual care, together with more pronounced improvement in secondary functional parameters and quality-of-life scores. Pharmacological optimization, reflected by higher uptake and intensification of guideline-directed medical therapy, also occurred more frequently in the structured pathway group. The integrated group, however, also had higher follow-up intensity, which limits causal interpretation of the observed between-group differences. Conclusions: In this real-world heart failure cohort, exposure to a structured care pathway combining repeated multimodal functional profiling with closer follow-up was associated with greater functional improvement and more intensive pharmacological optimization. These findings should be interpreted as pathway-level associations rather than proof that functional assessment alone drove benefit, and they require prospective validation.

## 1. Introduction

Chronic heart failure with reduced or mildly reduced ejection fraction remains a major cause of morbidity, hospitalization, and impaired quality of life despite substantial advances in pharmacological therapy [1,2]. Contemporary guideline-directed medical therapy has demonstrated clear benefits on survival and clinical outcomes, yet real-world data consistently show delayed initiation, incomplete titration, and underuse of combination regimens in routine practice [3]. A central challenge in everyday care is the lack of objective, reproducible functional assessment tools to support confident pharmacological optimization beyond symptoms and resting clinical parameters.

Heart failure (HF) is characterized by exercise intolerance arising from multifaceted pathophysiological mechanisms—including inadequate cardiac output, neurohormonal dysregulation, endothelial dysfunction, and peripheral hypoperfusion—resulting in diminished quality of life and adverse prognosis irrespective of left ventricular ejection fraction (LVEF). Cardiac rehabilitation (CR), especially aerobic and combined training [4], is recommended alongside optimized medical therapy for chronic HF patients per current guidelines, conferring diverse clinical, structural, and physiological benefits [5]

Cardiopulmonary exercise testing is the reference standard for integrative functional evaluation in heart failure, providing prognostically powerful measures of exercise capacity and ventilatory efficiency [6,7,8]. However, its limited availability and logistical complexity restrict its routine use in outpatient settings, resulting in a gap between optimal physiological assessment and real-world pharmacological decision-making [8].

The 6 min walk test represents a pragmatic alternative for functional assessment, reflecting submaximal exercise capacity closely aligned with daily activities [9]. Both baseline walk distance and longitudinal changes have been consistently associated with symptom burden, quality of life, hospitalization risk, and mortality in heart failure populations [10]. Importantly, improvements in 6 min walk distance have been shown to parallel therapeutic benefit in pharmacological trials, supporting its role as a responsive indicator of clinical change rather than a purely descriptive measure [11].

Additional information relevant to cardiopulmonary reserve can be derived from simple physiological and patient-reported parameters obtained during functional testing. Heart rate response and post-exercise recovery reflect chronotropic competence and autonomic function, both of which carry prognostic significance in heart failure [12,13]. Oxygen saturation response during exertion and perceived exertion further contextualize exercise limitation, while patient-reported quality of life assessed by the Minnesota Living with Heart Failure Questionnaire provides a validated measure of disease impact and treatment response [14].

Together, these measures capture core domains traditionally assessed by cardiopulmonary exercise testing, albeit at lower technical resolution, and may approximate its conceptual value when integrated into a structured assessment framework. Evidence remains limited, however, regarding whether such multimodal functional profiling can support more effective pharmacological optimization in real-world heart failure care.

Accordingly, the present study evaluates a structured care pathway centered on standardized functional assessment with the 6 min walk test and complementary physiological and patient-reported measures in patients with heart failure with reduced or mildly reduced ejection fraction undergoing outpatient cardiological rehabilitation and treatment. We aimed to determine whether exposure to this pathway was associated with greater functional improvement and more intensive optimization of guideline-directed medical therapy over six months compared with usual care.

## 2. Methods

### 2.1. Study Design and Population

This study was designed as a single-center, real-world retrospective cohort analysis conducted in a tertiary university-affiliated heart failure outpatient clinic. The study population consisted of adult patients with a documented diagnosis of chronic heart failure with reduced or mildly reduced ejection fraction who were followed longitudinally in routine clinical practice.

Inclusion criteria were age ≥ 18 years, a confirmed diagnosis of chronic heart failure with left ventricular ejection fraction below 50% documented by transthoracic echocardiography, clinical stability at the time of baseline functional assessment, and availability of complete functional and pharmacological data at baseline and at approximately six months. Heart failure with reduced ejection fraction was defined as a left ventricular ejection fraction < 40%, and heart failure with mildly reduced ejection fraction as an ejection fraction between 40% and 49%, in accordance with contemporary American Heart Association Joint Committee guideline definitions [15,16]. All included patients were required to be treated with at least one component of guideline-directed medical therapy at baseline and to have been followed in the outpatient setting for the entire observation period.

Exclusion criteria were heart failure with preserved ejection fraction, acute decompensated heart failure requiring hospitalization at the time of baseline assessment, significant non-cardiac conditions limiting exercise performance independently of heart failure, including advanced pulmonary disease, severe peripheral arterial disease, or neurologic or musculoskeletal disorders, inability to perform a standardized 6 min walk test, prior heart transplantation or mechanical circulatory support, and incomplete clinical, functional, or pharmacological documentation at follow-up.

After application of inclusion and exclusion criteria, eligible patients were retrospectively classified into two groups based on the documented care pathway implemented during initial examination and follow-up. Group allocation was determined exclusively by objective documentation in the electronic medical record and reflected real-world clinical practice rather than any investigator-assigned intervention. Because pathway assignment was not randomized, the analysis was designed to compare two care strategies as they were delivered in routine practice, with explicit acknowledgement of potential selection bias and residual confounding.

The integrated multimodal assessment group consisted of patients managed using a structured framework implemented through standardized functional profiling. In this group, patients followed a predefined outpatient evaluation schedule comprising a baseline visit, interim follow-up visits at approximately 6 and 12 weeks, and a comprehensive reassessment at six months. At baseline and at follow-up, a standardized multimodal functional assessment battery was systematically performed and documented, including the 6 min walk test, one-minute heart rate recovery (HRR), exertional oxygen saturation nadir, Borg perceived exertion score, and Minnesota Living with Heart Failure Questionnaire (MLHFQ) total score. Based on these measures, patients were categorized into three functional severity tiers using predefined thresholds reflecting published prognostic data: mild impairment (6MWD ≥ 350 m, HRR ≥ 15 bpm, MLHFQ ≤ 45), moderate impairment (6MWD 250–349 m, HRR 10–14 bpm, MLHFQ 46–65), and severe impairment (6MWD < 250 m, HRR < 10 bpm, MLHFQ > 65) [17,18]. When parameters were discordant, classification was determined by the most severe abnormality.

Treatment intensification was mapped to functional tier unless contraindicated. In mild impairment, focus was placed on up-titration toward target doses of existing guideline-directed medical therapy (GDMT). In moderate impairment, initiation of missing GDMT classes and structured dose escalation were prioritized. In severe impairment, accelerated optimization, closer follow-up, and consideration of advanced heart failure referral were implemented. Treatment escalation remained clinician-led rather than algorithmically mandatory, and blood pressure, heart rate, renal function, potassium, symptom burden, and overall tolerability were considered at each decision point. To quantify pharmacological adjustment, a Treatment Modification Intensity Index was defined, assigning weighted points to dose escalation, initiation of new GDMT classes, and multiple changes per visit, enabling categorization into low, moderate, or high treatment intensity. The usual care group did not undergo formalized tier classification or predefined escalation mapping.

The usual care group was managed according to contemporary standard clinical practice and represents the routine care pathway used in the outpatient heart failure clinic during the study period. Patients in this group underwent a baseline clinical evaluation and a follow-up assessment at approximately six months, with additional contacts performed as clinically indicated. These contacts included both in-person visits and telemedicine consultations, reflecting real-world practice patterns. Treatment adjustments in the usual care group were based on symptoms, functional status as reported by the patient, blood pressure, heart rate, laboratory parameters, and clinician judgment, in accordance with guideline recommendations. Pharmacological changes, including initiation or up-titration of GDMT components, were therefore not withheld in the control group but were implemented without the support of a standardized, repeated multimodal functional assessment package.

Importantly, both groups had access to the same clinical resources, treating physicians, and guideline-based therapeutic options, including telemedicine follow-up when appropriate. The primary distinction between groups was the systematic use of a structured, repeated multimodal functional assessment framework together with prespecified interim reassessment in the integrated group, as opposed to symptom-driven and clinician-directed management in the usual care group. For this reason, the present analysis addresses a composite care pathway effect rather than the isolated mechanistic effect of functional assessment alone.

Regarding evaluation control, the 6 min walk test was performed in accordance with established protocols in a flat, indoor corridor, with standardized instructions and encouragement. Total distance walked in meters was recorded as the primary functional measure. Resting heart rate was measured after a standardized seated rest period prior to testing, and peak heart rate during the test was recorded. Heart rate recovery was calculated as the difference between peak heart rate and heart rate measured one minute after test completion. Peripheral oxygen saturation was monitored continuously using pulse oximetry, and the lowest saturation value recorded during exertion was used for analysis. Perceived exertion was assessed immediately after completion of the test using the Borg 6–20 scale. Health-related quality of life was evaluated using the Minnesota Living with Heart Failure Questionnaire, with total scores ranging from 0 to 105, lower scores indicating better perceived health status.

Pharmacological therapy was reviewed at each outpatient visit. Guideline-directed medical therapy was defined according to European Society of Cardiology recommendations and included renin–angiotensin system inhibitors or angiotensin receptor–neprilysin inhibitors, evidence-based beta-blockers, mineralocorticoid receptor antagonists, and sodium–glucose cotransporter 2 inhibitors [19]. GDMT intensity was quantified as the number of these therapeutic pillars prescribed at baseline and at six months. Treatment intensification was defined as initiation of at least one additional GDMT class or documented up-titration toward guideline-recommended or maximally tolerated doses during the follow-up period.

### 2.2. Study Objectives

The primary endpoint was the absolute change in 6 min walk distance from baseline to six months. Secondary endpoints included changes in heart rate recovery, resting heart rate, oxygen saturation nadir during exertion, Borg perceived exertion score, and Minnesota Living with Heart Failure Questionnaire total score. Changes in GDMT intensity and the proportion of patients undergoing treatment intensification were analyzed as secondary therapeutic outcomes. Heart failure-related hospitalization within the six-month follow-up period was recorded as an exploratory clinical endpoint. Follow-up echocardiography and NYHA reclassification were not uniformly available at six months in both pathways as part of routine care and therefore were not retained as prespecified longitudinal endpoints.

### 2.3. Data Extraction and Statistical Analysis

Clinical, functional, and pharmacological data were extracted retrospectively from the hospital’s electronic medical record system and integrated digital cardiology database. Functional test results, questionnaire scores, medication prescriptions, and follow-up visit documentation were retrieved using structured query tools and manual verification by two independent investigators to ensure data completeness and accuracy. Any discrepancies were resolved by consensus review of the original source documents.

The structured assessment pathway was designed to reflect principles commonly used in cardiovascular rehabilitation programs, where repeated functional evaluation is used to guide both exercise prescription and optimization of medical therapy.

Data management was performed using Microsoft Excel 2016 (Microsoft Corp., Redmond, WA, USA) [20], and statistical analysis was performed using IBM SPSS Statistics for Windows, version 26.0 (IBM Corp., Armonk, NY, USA) [21]. Continuous variables were assessed for normality and are presented as mean values with standard deviations or as medians with interquartile ranges, as appropriate. Categorical variables are expressed as counts and percentages. Between-group comparisons were conducted using independent-sample *t* tests or Mann–Whitney U tests for continuous variables and chi-square or Fisher’s exact tests for categorical variables. Within-group changes from baseline to follow-up were evaluated using paired-sample tests. Because only two observation time points were available, the between-group comparison of change scores is equivalent to the group-by-time interaction term from a repeated-measures model and was reported as such for interpretive clarity. An exploratory multivariable linear regression analysis was used to examine the association between pathway exposure and change in 6 min walk distance, adjusting for relevant baseline clinical characteristics and GDMT intensity. Visit frequency was not entered into the primary model because it was intrinsically linked to pathway assignment and would therefore not permit clean separation of pathway content from pathway intensity. A two-sided *p* value < 0.05 was considered statistically significant.

### 2.4. Ethical Considerations

The study was conducted in accordance with the Declaration of Helsinki [22] and complied with the European Union General Data Protection Regulation (GDPR) [23]. The research protocol was approved by the local institutional ethics committee (Ethics Approval no. 1454/19 February 2026). Given the retrospective, observational design and the exclusive use of anonymized data extracted from routine clinical records, the requirement for written informed consent was formally waived by the ethics board.

## 3. Results

### 3.1. Study Population and Group Allocation

A total of 312 consecutive patients with a diagnosis of chronic heart failure were initially identified from the electronic medical record system of the tertiary university-affiliated outpatient clinic during the study period. After detailed chart review, 62 patients were excluded due to predefined criteria. These included heart failure with preserved ejection fraction, acute decompensated heart failure at the time of baseline assessment, inability to perform standardized functional testing due to non-cardiac limitations, and incomplete functional or follow-up data. The final study cohort therefore consisted of 250 patients with heart failure with reduced or mildly reduced ejection fraction who fulfilled all inclusion criteria and had complete baseline and six-month follow-up information available for analysis.

Of these, 130 patients were managed using the integrated multimodal functional assessment pathway, while 120 patients received usual care. No patients were excluded after group allocation due to missing follow-up data. The median follow-up duration was 6.2 months and did not differ between groups.

Baseline demographic and clinical characteristics were comparable between the two groups. Mean age was 65.4 ± 9.8 years in the integrated assessment group and 66.1 ± 10.2 years in the usual care group (*p* = 0.58). Male sex predominated in both cohorts (61.5% vs. 60.0%, *p* = 0.81). Heart failure with reduced ejection fraction accounted for the majority of cases in both groups (72.3% in the integrated group vs. 70.8% in usual care, *p* = 0.79), with the remainder classified as heart failure with mildly reduced ejection fraction. Baseline left ventricular ejection fraction was similar between groups (33.6 ± 7.4% vs. 34.1 ± 7.1%, *p* = 0.61). The distribution of New York Heart Association functional class did not differ significantly, with approximately half of patients in both groups classified as NYHA class III.

Comorbidities were evenly distributed. The prevalence of diabetes mellitus, chronic kidney disease stage ≥ 3, atrial fibrillation, and ischemic heart disease was similar across groups, with no statistically significant differences observed. Baseline pharmacological treatment reflected contemporary guideline-directed medical therapy and was well balanced. The mean number of GDMT pillars at baseline was 2.1 ± 0.9 in the integrated assessment group and 2.0 ± 0.8 in the usual care group (*p* = 0.42), with comparable prescription rates for renin–angiotensin system inhibitors or angiotensin receptor–neprilysin inhibitors, beta-blockers, mineralocorticoid receptor antagonists, and sodium–glucose cotransporter 2 inhibitors.

Baseline demographic, clinical, functional, and pharmacological characteristics of the study population stratified by management strategy are summarized in Table 1.

### 3.2. Baseline Functional and Patient-Reported Status

At study entry, functional capacity assessed by the 6 min walk test was similarly impaired in both groups. Mean baseline walk distance was 318 ± 92 m in the integrated assessment group and 323 ± 88 m in the usual care group (*p* = 0.68). Resting heart rate prior to testing averaged 79 ± 13 beats per minute in the integrated group and 78 ± 12 beats per minute in usual care (*p* = 0.54). One-minute heart rate recovery was reduced in both cohorts, with mean values of 13.9 ± 6.1 beats per minute and 14.2 ± 5.8 beats per minute, respectively (*p* = 0.71). Oxygen saturation nadir during exertion was comparable between groups (92.4 ± 3.1% vs. 92.6 ± 3.0%, *p* = 0.63).

**Figure 1:**
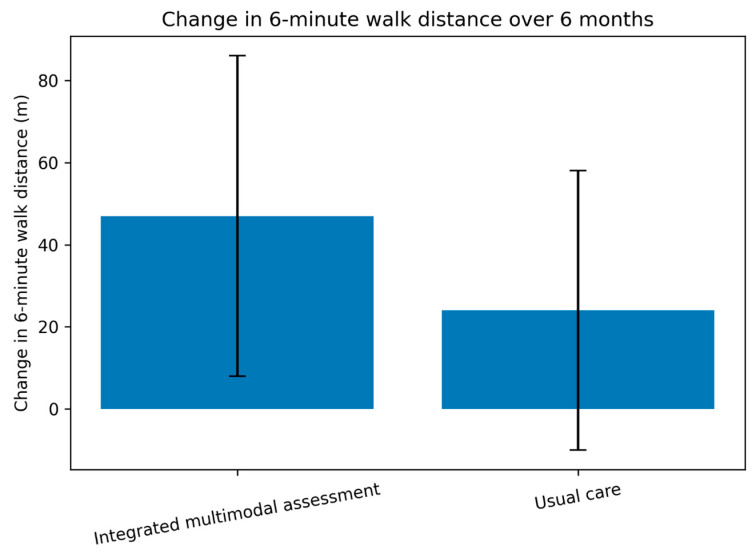
Comparison of change in 6 min walk distance from baseline to six months in patients managed using the integrated multimodal functional assessment pathway and those receiving usual care. Bars represent mean values, and error bars indicate standard deviation. 6MWD, 6 min walk distance.

Changes in 6 min walk distance from baseline to six months according to management strategy are shown in Figure 1.

Perceived exertion at the end of the walk test was high in both cohorts, with mean Borg scores of 14.1 ± 2.4 in the integrated assessment group and 13.9 ± 2.5 in the usual care group (*p* = 0.48). Quality of life assessed by the Minnesota Living with Heart Failure Questionnaire revealed substantial impairment at baseline, with mean total scores of 54.6 ± 16.8 and 53.9 ± 17.2, respectively (*p* = 0.77). These findings indicate a closely matched functional and symptomatic profile at baseline.

**Figure 2:**
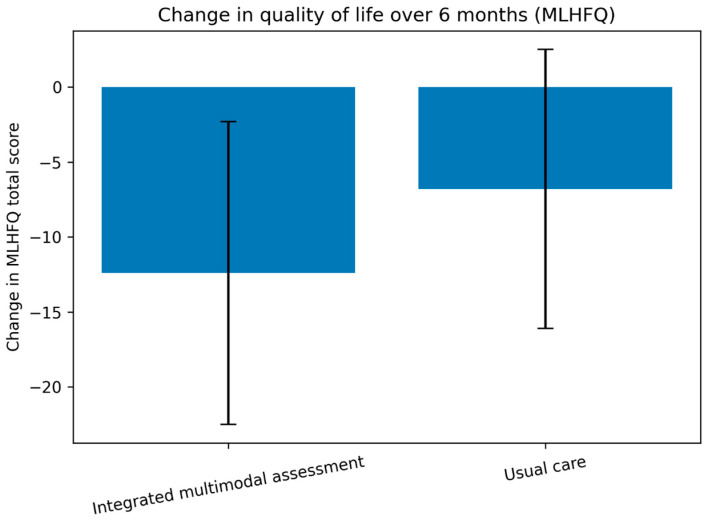
Change in Minnesota Living with Heart Failure Questionnaire total score from baseline to six months according to management strategy. Negative values indicate improvement in health-related quality of life. MLHFQ, Minnesota Living with Heart Failure Questionnaire.

Between-group differences in changes in health-related quality of life over the six-month follow-up period are presented in Figure 2.

Changes in functional capacity, physiological response to exertion, and quality of life over the six-month follow-up period are presented in Table 2.

### 3.3. Follow-Up Intensity and Pharmacological Optimization

During the six-month follow-up period, patients in the integrated multimodal assessment group attended a median of four structured clinical encounters, including baseline, two interim visits, and the six-month reassessment. Patients in the usual care group had a median of two documented encounters, typically comprising a baseline visit and a follow-up visit, supplemented by telemedicine or additional in-person contacts when clinically indicated. Importantly, access to pharmacological therapy, laboratory testing, and telemedicine follow-up was similar in both groups, but the difference in contact frequency remains an important potential confounder when interpreting pathway-associated benefit.

Treatment intensification occurred significantly more frequently in the integrated assessment group. At least one GDMT pillar was initiated or up-titrated in 72.3% of patients in the integrated group compared with 49.2% in the usual care group (*p* < 0.001). By six months, the mean number of GDMT pillars increased to 2.9 ± 0.8 in the integrated group, compared with 2.4 ± 0.9 in the usual care group (*p* < 0.001). The proportion of patients receiving three or more GDMT pillars at follow-up was 64.6% in the integrated group versus 41.7% in usual care (*p* < 0.001).

### 3.4. Functional Outcomes at Six Months

At six months, functional capacity improved in both groups, but the magnitude of improvement was significantly greater in patients managed with the integrated multimodal assessment pathway. Mean 6 min walk distance increased by 47 ± 39 m in the integrated group and by 24 ± 34 m in the usual care group (between-group *p* < 0.001). Within-group paired analyses confirmed significant improvement from baseline to six months in both cohorts (both *p* < 0.001). The absolute between-group difference in change was therefore 23 m, which favored the integrated pathway but remained below the commonly cited 25–35 m range for minimal clinically important difference in chronic heart failure. Clinically meaningful improvement, defined as an increase of at least 30 m, was achieved in 61.5% of patients in the integrated group and 38.3% of patients in usual care (*p* < 0.001) (Table 2), although this secondary responder analysis should be interpreted in the context of the borderline primary between-group effect size.

Heart rate recovery at one minute improved by 4.3 ± 3.9 beats per minute in the integrated group, compared with 2.1 ± 3.5 beats per minute in usual care (*p* = 0.002). Resting heart rate decreased modestly in both groups, with a greater reduction observed in the integrated group (−6.1 ± 7.8 vs. −3.4 ± 6.9 beats per minute, *p* = 0.01). Oxygen saturation nadir during exertion increased by 1.3 ± 1.8% in the integrated group and by 0.6 ± 1.6% in the usual care group (*p* = 0.02). Borg perceived exertion scores decreased by 1.6 ± 1.4 points in the integrated group, compared with 0.9 ± 1.3 points in usual care (*p* = 0.01).

Quality of life improved in both cohorts, but more substantially in patients undergoing structured functional profiling. MLHFQ total score decreased by 12.4 ± 10.1 points in the integrated assessment group, compared with a reduction of 6.8 ± 9.3 points in the usual care group (*p* < 0.001).

### 3.5. Association Between Functional Assessment Pathway and Improvement in Walk Distance

In an exploratory multivariable linear regression analysis adjusting for age, sex, baseline left ventricular ejection fraction, baseline walk distance, NYHA class, and baseline GDMT intensity, exposure to the integrated multimodal assessment pathway was associated with greater improvement in 6 min walk distance (β = +18.6 m, 95% CI 9.4–27.8, *p* < 0.001). Treatment intensification was also associated with walk distance improvement (β = +12.3 m, 95% CI 4.1–20.5, *p* = 0.004). This model did not adjust for visit frequency, which differed between pathways and should therefore be considered a major source of residual confounding rather than evidence of an isolated independent functional assessment effect.

Changes in guideline-directed medical therapy intensity and treatment intensification rates during follow-up are summarized in Table 3.

### 3.6. Clinical Outcomes

Heart failure-related hospitalization within six months occurred in 11.5% of patients in the integrated assessment group and 17.5% of patients in the usual care group (*p* = 0.17). Although numerically lower in the integrated group, this difference did not reach statistical significance and was considered exploratory.

## 4. Discussion

### 4.1. Principal Findings

In this real-world retrospective cohort of patients with heart failure with reduced or mildly reduced ejection fraction, exposure to a structured care pathway implemented through repeated multimodal functional profiling was associated with greater improvement in functional capacity over six months, with the 6 min walk test serving as the primary anchor outcome. However, the between-group difference in change in walk distance was 23 m, which is below the lower bound of the commonly cited 25–35 m range for minimal clinically important difference in chronic heart failure [24,25,26,27]. The primary endpoint should therefore be interpreted cautiously: it was statistically robust but clinically modest at the between-group level, and the more favorable ≥ 30 m responder proportion should be regarded as supportive secondary context rather than as a way to rescue the primary effect size.

Beyond the primary endpoint, the integrated assessment pathway was associated with concordant improvement across complementary functional domains, including heart rate recovery, exertional oxygen saturation, perceived exertion, and disease-specific quality of life. This multidimensional response supports the internal coherence of the model, in which objective performance measures and patient-reported outcomes move in parallel under more structured therapeutic guidance. Such convergence is clinically relevant, as discordance between physiologic improvement and patient-reported benefit has been identified as a marker of suboptimal treatment alignment in chronic heart failure [28,29,30,31].

A second principal finding was the higher intensity of pharmacological optimization observed in patients managed using the structured pathway. Despite the strong evidence base supporting guideline-directed medical therapy, contemporary registry and implementation studies continue to show underuse and delayed up-titration of recommended drug classes in routine practice, particularly among older patients and those with greater clinical complexity [32,33,34]. In the present study, however, more intensive pharmacological optimization cannot be attributed solely to the functional assessment battery, because the pathway also entailed more frequent contact with the treating team. The observed benefit is therefore best interpreted as the effect of a bundled strategy that combined repeated reassessment, closer follow-up, and more opportunities for treatment adjustment [35].

A third important aspect of the results relates to how structured assessment is conceptualized in contemporary heart failure management. While cardiopulmonary exercise testing remains the physiological reference standard, its limited availability constrains routine use as a decision-support tool. Recent position papers and implementation-focused analyses have emphasized the need for scalable strategies that translate standardized evaluation principles into everyday practice without requiring maximal exercise testing or specialized infrastructure [36,37]. Even so, the present study should not be read as proof that the multimodal functional package itself was the sole active ingredient. Rather, it suggests that a pragmatic, structured pathway in which feasible functional metrics are repeatedly integrated into follow-up may support more active care delivery.

### 4.2. The 6-Minute Walk Test as a Functional Anchor

Positioning the 6 min walk test as the central functional endpoint within the framework can be justified from several complementary perspectives, each emphasizing a different dimension of its clinical relevance while acknowledging its limitations. First, from a physiological standpoint, the 6 min walk test captures integrated submaximal exercise capacity, reflecting the combined effects of cardiac output, chronotropic response, peripheral oxygen extraction, and skeletal muscle conditioning [9,10]. Although it does not provide the mechanistic granularity as other standardized models, such as cardiopulmonary exercise testing (CPET) variables such as peak oxygen uptake or ventilatory efficiency, several studies have demonstrated moderate correlations between 6 min walk distance and peak VO_2_ in patients with systolic heart failure, particularly in populations with moderate-to-severe functional limitation [18,27]. This relationship supports the interpretation of the 6 min walk test as a pragmatic surrogate for global exercise capacity when maximal testing is not feasible.

A second interpretative framework emphasizes the ecological validity of the 6 min walk test. Unlike CPET, which assesses performance under controlled laboratory conditions and maximal effort, the 6 min walk test reflects sustained submaximal exertion that more closely resembles activities of daily living [9]. This characteristic may explain why changes in walk distance are often more tightly aligned with patient-reported symptoms and perceived functional improvement than changes in maximal exercise parameters [38]. Several longitudinal studies have shown that improvements in 6 min walk distance parallel changes in daily activity levels and health-related quality of life, even in the absence of large shifts in peak exercise capacity [11,24,38,39,40]. From this perspective, the 6 min walk test may be particularly well suited to guide outpatient pharmacological optimization aimed at improving day-to-day functioning rather than maximal performance.

A third way of viewing the role of the 6 min walk test relates to its responsiveness to therapeutic intervention. In contrast to some other objective parameters that may change slowly or require substantial physiological remodeling, the 6 min walk distance has demonstrated sensitivity to short- and medium-term changes induced by pharmacological therapy, exercise-based rehabilitation, and combined management strategies [11,24,26]. Meta-analyses and post hoc analyses of heart failure trials have shown that improvements in walk distance can be detected within months of treatment initiation or intensification and are associated with downstream reductions in hospitalization risk [35,41,42,43]. This responsiveness makes the test particularly attractive for longitudinal monitoring in settings where treatment adjustments occur over relatively short intervals, as in the present study.

At the same time, it is important to acknowledge the limitations of the 6 min walk test within our framework. The test is influenced by patient motivation, musculoskeletal comorbidity, and learning effects, and it lacks the ability to disentangle central from peripheral contributors to exercise limitation [44]. Moreover, its prognostic value appears attenuated in patients with milder symptoms, where ceiling effects may reduce discrimination [8,18,36]. These limitations underscore why the present study did not rely on walk distance in isolation but integrated it with complementary physiological parameters, including heart rate dynamics and oxygen saturation response, as well as patient-reported quality of life. Such integration aligns with contemporary recommendations advocating for multidimensional functional assessment rather than reliance on a single metric.

### 4.3. Clinical Implications

The findings of the present study have several practical implications for the outpatient management of chronic heart failure with reduced or mildly reduced ejection fraction. First, they suggest that structured functional assessment can be incorporated into routine care without reliance on advanced cardiopulmonary exercise testing infrastructure. By centering follow-up around reproducible, low-burden functional measures such as the 6 min walk test and complementary physiological and patient-reported parameters, clinicians may obtain longitudinal signals that can support pharmacological decision-making and rehabilitation planning [45,46].

Second, the present results should be interpreted alongside other structured GDMT implementation models. Diederich et al. described the prospective multidisciplinary HF-Optimize clinic, which used six visits over 12 weeks and reported improvement in GDMT use together with better 6 min walk distance, quality of life, and ejection fraction in adults with HFrEF [47]. Other contemporary studies and reviews by Straw, Khan, McCullough, and Van der Linden further emphasize that systematic GDMT implementation, therapeutic inertia, frailty, and patient complexity materially influence how much benefit is achieved in routine practice [48,49,50,51]. Taken together, these studies support the value of organized heart failure pathways, but they also reinforce that structured contact intensity and implementation strategy are likely to matter as much as the specific bedside metrics being collected.

Third, pooling HFrEF and HFmrEF requires caution. The 2021 ESC guideline distinguished these phenotypes for several drug classes, and the 2023 focused update further strengthened the evidence base for selected therapies in HFmrEF, especially SGLT2 inhibition [19,52]. We retained both phenotypes because the present study addressed a pragmatic pathway question in ambulatory patients with LVEF < 50% rather than a drug-class efficacy question, and because observational data suggest that HFmrEF often resembles HFrEF clinically and may derive benefit from GDMT across the LVEF < 50% spectrum [48]. Nevertheless, the current results should be interpreted as pooled LVEF < 50% findings rather than phenotype-specific estimates.

### 4.4. Strengths, Limitations and Future Directions

This study benefits from a real-world design, inclusion of a contemporary outpatient heart failure population, and evaluation of a pragmatic, scalable functional assessment strategy aligned with current guideline-based care. The integrated use of objective functional testing and patient-reported outcomes reflects a multidimensional approach relevant to routine clinical practice.

An additional perspective for interpreting the present findings is through the lens of cardiovascular rehabilitation. Structured functional reassessment is a core component of rehabilitation-based heart failure care, allowing iterative adjustment of exercise intensity and pharmacological therapy. The present model extends this principle into routine outpatient management by using repeated functional profiling as a shared decision-support tool between clinician and patient [4,5].

Several limitations should be acknowledged. The retrospective, non-randomized design precludes causal inference, and residual confounding related to clinician behavior, patient engagement, or unmeasured clinical factors cannot be excluded. Most importantly, the integrated pathway involved more frequent clinical contacts than usual care, which may itself explain part or even most of the observed differences in GDMT optimization and functional outcomes. Accordingly, the results should be interpreted as reflecting a composite care pathway rather than the isolated effect of multimodal functional assessment. Group allocation was based on documented care pathways, introducing potential selection bias. Functional evaluation relied on submaximal testing and did not include cardiopulmonary exercise testing, limiting mechanistic interpretation. Pharmacological optimization was assessed using therapy intensity rather than achieved target doses. Follow-up echocardiography and NYHA reclassification were not uniformly available, and phenotype-specific subgroup analyses for HFrEF versus HFmrEF were not available in a sufficiently standardized form for robust inference. Finally, the single-center setting and relatively short follow-up limit generalizability and preclude definitive conclusions regarding hard clinical outcomes.

Future studies should prospectively evaluate structured care pathways that combine repeated functional assessment with explicit follow-up schedules and GDMT optimization strategies, ideally using pragmatic randomized or carefully controlled prospective designs to separate the contribution of functional profiling from the contribution of contact intensity. Such work should also prespecify phenotype-restricted analyses, include standardized follow-up echocardiography and NYHA reassessment, and build on existing implementation models such as multidisciplinary GDMT clinics [47]. Integration of standardized functional testing into telemedicine and hybrid outpatient care pathways remains particularly relevant as care models continue to evolve.

## 5. Conclusions

In a real-world outpatient cohort of patients with heart failure with reduced or mildly reduced ejection fraction, exposure to a structured care pathway combining repeated functional assessment with closer follow-up was associated with greater functional improvement, better health-related quality of life, and more intensive optimization of guideline-directed medical therapy compared with usual care. The primary between-group gain in 6 min walk distance was modest, and the study design does not allow attribution of benefit to functional assessment alone. These findings should therefore be viewed as hypothesis-generating support for structured pathway-based management rather than definitive proof of a stand-alone decision-support effect of the multimodal assessment package.

## Figures and Tables

**Table 1:** Baseline characteristics of the study population.

Characteristic	Integrated Group (*n* = 130)	Usual Care Group (*n* = 120)	*p* Value
Age, years (mean ± SD)	65.4 ± 9.8	66.1 ± 10.2	0.58
Male sex, *n* (%)	80 (61.5)	72 (60.0)	0.81
Heart failure phenotype, *n* (%)			
HFrEF (<40%)	94 (72.3)	85 (70.8)	0.79
HFmrEF (40–49%)	36 (27.7)	35 (29.2)	
Left ventricular ejection fraction, % (mean ± SD)	33.6 ± 7.4	34.1 ± 7.1	0.61
NYHA functional class, *n* (%)			
Class II	62 (47.7)	58 (48.3)	0.92
Class III	58 (44.6)	52 (43.3)	
Class IV	10 (7.7)	10 (8.4)	
Comorbidities, *n* (%)			
Diabetes mellitus	42 (32.3)	38 (31.7)	0.92
Chronic kidney disease (≥stage 3)	34 (26.2)	30 (25.0)	0.83
Atrial fibrillation	38 (29.2)	34 (28.3)	0.88
Ischemic heart disease	74 (56.9)	66 (55.0)	0.76
Functional parameters (baseline)			
6 min walk distance, m	318 ± 92	323 ± 88	0.68
Resting heart rate, bpm	79 ± 13	78 ± 12	0.54
Heart rate recovery (1 min), bpm	13.9 ± 6.1	14.2 ± 5.8	0.71
Oxygen saturation nadir during exertion, %	92.4 ± 3.1	92.6 ± 3.0	0.63
Borg perceived exertion score	14.1 ± 2.4	13.9 ± 2.5	0.48
Quality of life (MLHFQ total score)	54.6 ± 16.8	53.9 ± 17.2	0.77
Pharmacological therapy			
Number of GDMT pillars (mean ± SD)	2.1 ± 0.9	2.0 ± 0.8	0.42
≥3 GDMT pillars, *n* (%)	46 (35.4)	40 (33.3)	0.74

Baseline demographic, clinical, functional, and pharmacological characteristics of patients managed using the integrated multimodal functional assessment pathway compared with usual care. Values are presented as mean ± standard deviation or number (percentage). HFrEF, heart failure with reduced ejection fraction; HFmrEF, heart failure with mildly reduced ejection fraction; NYHA, New York Heart Association; MLHFQ, Minnesota Living with Heart Failure Questionnaire; GDMT, guideline-directed medical therapy.

**Table 2:** Functional and quality-of-life outcomes at baseline and 6-month follow-up.

Outcome	Context	Integrated Group (*n* = 130)	Usual Care Group (*n* = 120)	*p* Value (Between Groups)
6 min walk distance, m	Baseline	318 ± 92	323 ± 88	0.68
	6 months	365 ± 95	347 ± 91	0.04
	Change (Δ)	+47 ± 39	+24 ± 34	<0.001
	≥30 m improvement, *n* (%)	80 (61.5)	46 (38.3)	<0.001
Resting heart rate, bpm	Baseline	79 ± 13	78 ± 12	0.54
	6 months	73 ± 12	75 ± 12	0.18
	Change (Δ)	−6.1 ± 7.8	−3.4 ± 6.9	0.01
Heart rate recovery (1 min), bpm	Baseline	13.9 ± 6.1	14.2 ± 5.8	0.71
	6 months	18.2 ± 6.4	16.3 ± 6.1	0.03
	Change (Δ)	+4.3 ± 3.9	+2.1 ± 3.5	0.002
Oxygen saturation nadir, %	Baseline	92.4 ± 3.1	92.6 ± 3.0	0.63
	6 months	93.7 ± 2.8	93.2 ± 2.9	0.21
	Change (Δ)	+1.3 ± 1.8	+0.6 ± 1.6	0.02
Borg perceived exertion score	Baseline	14.1 ± 2.4	13.9 ± 2.5	0.48
	6 months	12.5 ± 2.2	13.0 ± 2.3	0.12
	Change (Δ)	−1.6 ± 1.4	−0.9 ± 1.3	0.01
MLHFQ total score	Baseline	54.6 ± 16.8	53.9 ± 17.2	0.77
	6 months	42.2 ± 15.4	47.1 ± 16.6	0.03
	Change (Δ)	−12.4 ± 10.1	−6.8 ± 9.3	<0.001

Changes in functional capacity, physiological parameters, and quality of life from baseline to six months in patients managed using the integrated multimodal functional assessment pathway compared with usual care. Values are presented as mean ± standard deviation or number (percentage). Positive change indicates improvement for 6 min walk distance, heart rate recovery, and oxygen saturation nadir, while negative change indicates improvement for resting heart rate, Borg perceived exertion score, and MLHFQ total score. Between-group *p* values refer to the comparison of follow-up values or change scores as indicated; within-group paired comparisons for all reported change measures were statistically significant in both groups (all *p* < 0.001). MLHFQ, Minnesota Living with Heart Failure Questionnaire.

**Table 3:** Guideline-directed medical therapy optimization and association with functional improvement.

Variable	Context	Integrated Group (*n* = 130)	Usual Care Group (*n* = 120)	*p* Value
GDMT intensity	GDMT pillars at baseline (mean ± SD)	2.1 ± 0.9	2.0 ± 0.8	0.42
	GDMT pillars at 6 months (mean ± SD)	2.9 ± 0.8	2.4 ± 0.9	<0.001
	Change in GDMT pillars (Δ)	+0.8 ± 0.6	+0.4 ± 0.6	<0.001
	≥3 GDMT pillars at 6 months, *n* (%)	84 (64.6)	50 (41.7)	<0.001
Treatment intensification	Any GDMT initiation or up-titration, *n* (%)	94 (72.3)	59 (49.2)	<0.001
	≥2 GDMT changes during follow-up, *n* (%)	38 (29.2)	18 (15.0)	0.006
Multivariable linear regression for Δ6MWD	Integrated assessment pathway (yes vs. no)	β = +18.6 m (95% CI 9.4–27.8)	—	<0.001
	GDMT intensification (yes vs. no)	β = +12.3 m (95% CI 4.1–20.5)	—	0.004
	Baseline 6MWD (per 10 m increase)	β = −1.8 m (95% CI −2.7 to −0.9)	—	<0.001
	Age (per 5-year increase)	β = −3.2 m (95% CI −6.1 to −0.3)	—	0.03

Optimization of guideline-directed medical therapy during follow-up and associations with improvement in 6 min walk distance. Values are presented as mean ± standard deviation, number (percentage), or regression coefficients with 95% confidence intervals. The exploratory multivariable linear regression model was adjusted for age, sex, baseline left ventricular ejection fraction, NYHA functional class, baseline 6 min walk distance, and baseline GDMT intensity, but not for visit frequency, which was intrinsically linked to pathway assignment. GDMT, guideline-directed medical therapy; 6MWD, 6 min walk distance; CI, confidence interval.

## Data Availability

All the data and materials will be made available on written requests.

## Abbreviations

6MWD	6 min walk distance
6MWT	6 min walk test
ARNI	Angiotensin receptor–neprilysin inhibitor
CPET	Cardiopulmonary exercise testing
GDMT	Guideline-directed medical therapy
HF	Heart failure
HFmrEF	Heart failure with mildly reduced ejection fraction
HFrEF	Heart failure with reduced ejection fraction
LVEF	Left ventricular ejection fraction
HR	Heart rate
HRR	Heart rate recovery
MLHFQ	Minnesota Living with Heart Failure Questionnaire
NYHA	New York Heart Association
QoL	Quality of life
